# Second opinions and pathological review impact the clinical management of patients with muscle-invasive bladder cancer

**DOI:** 10.1007/s00345-025-05861-5

**Published:** 2025-10-09

**Authors:** Takashi Kawahara, Satoshi Nitta, Masanobu Shiga, Yoshiyuki Nagumo, Shuya Kandori, Hiromitsu Negoro, Noriaki Sakamoto, Daisuke Matsubara, Hiroyuki Nishiyama

**Affiliations:** 1 Department of Urology, Faculty of Medicine, University of Tsukuba, 1-1-1 Tennodai, Tsukuba, Ibaraki 305-8575 Japan; 2 Department of Diagnostic Pathology, University of Tsukuba, 1-1-1 Tennodai, Ibaraki 305-8575 Tsukuba, Japan

**Keywords:** Urinary bladder neoplasms, Urinary bladder, Tumor staging, Muscle invasive bladder cancer

## Abstract

**Purpose:**

Studies evaluating inter-institutional discrepancies in muscle-invasive bladder cancer diagnosis, particularly when radical cystectomy is recommended, are scarce. In this study, we aimed to examine the inter-institutional concordance rate of pathological stages in patients diagnosed with muscle-invasive bladder cancer.

**Methods:**

We reviewed tissue samples from patients pathologically diagnosed with muscle-invasive bladder cancer at other hospitals who subsequently sought a second opinion at our hospital between January 1, 2013 and December 31, 2023. Data were acquired retrospectively by retrieving clinical data from medical records. We investigated the inter-institutional concordance rate of pathological stages assigned to each patient and examined the tumor characteristics and prognoses of patients with pathological stage discrepancies.

**Results:**

Of the 170 patients evaluated, 22 (12.9%) were downstaged upon pathological examination at our hospital. The tumor characteristics of stage discrepancy cases were small tumor size, solitary lesions, and vesical imaging reporting data system score < 3. Eleven patients were treated for non-muscle-invasive bladder cancer based on the re-evaluation findings. Eight patients had no evidence of disease, two patients had Ta bladder recurrence, and one patient had lung metastasis despite no bladder recurrence.

**Conclusion:**

This study highlights the importance of pathological re-evaluation of patients diagnosed with muscle-invasive bladder cancer. The significant discrepancy rate and its impact on the treatment decisions are noteworthy. Standardization of diagnostic practices is essential to mitigate variability among pathologists and facilities and to ensure optimal care for patients with muscle-invasive bladder cancer.

## Introduction

Bladder cancer affects approximately 614,000 individuals each year. An estimated 220,000 bladder cancer-related deaths occurred in 2022, making it the 9th most common cancer in terms of new cases and 13th leading cause of cancer-related deaths worldwide [1]. Bladder cancer is clinically classified into non-muscle-invasive bladder cancer (NMIBC) or muscle-invasive bladder cancer (MIBC), each requiring a distinct treatment approach [2–4]. NMIBC is managed by transurethral resection (TUR) of the bladder tumor (TURBT), followed by intravesical therapy, whereas MIBC often requires more invasive treatments, such as radical cystectomy or radiation therapy, in the absence of metastasis. Thus, the accurate assessment of muscle invasion is clinically important.

NMIBC and MIBC can be differentiated based on the presence or absence of invasion into the muscularis propria, which depends on the pathological diagnosis [5]. Although magnetic resonance imaging (MRI) plays a complementary role in localizing bladder tumor staging [6], the pathological diagnosis is ultimately achieved through TURBT. Accurate staging of NMIBC and MIBC requires tumor resection, including the muscularis propria, because failure to do so may result in understaging or residual tumor risk, as indicated by clinical guidelines [2, 4].

Discrepancies in grade and stage diagnoses, even when the muscularis propria is sampled, have been attributed to tumor fragmentation or artifacts from thermal cauterization during TURBT [7]. Inter-pathologist discrepancies in interpretations have been reported mainly in NMIBC [8–14]. Studies evaluating the inter-institutional discrepancies in MIBC diagnosis, particularly when radical cystectomy is recommended, are scarce. Moreover, whether the disparities in the pathological diagnosis of MIBC have diminished since the strong recommendation for muscularis propria sampling remains unclear.

The primary objective of this study was to assess the concordance rate of pathological stages among patients with MIBC diagnosed outside our hospital. The secondary objective was to evaluate the rates of treatment changes and prognosis based on pathological diagnoses at our institution.

## Materials and methods

We reviewed the clinical records of patients diagnosed with MIBC by local pathologists according to local practice at various hospitals who subsequently sought a second opinion at our hospital between January 1, 2013 and December 31, 2023. Patients who desired evaluation and treatment at our hospital and underwent re-evaluation of their pathological slides were included. Patients who did not desire evaluation or underwent pathological re-evaluation at our hospital were excluded.

Clinical data from medical records were retrospectively reviewed. Data on the number of tumors, tumor size, tumor morphology, preoperative MRI findings, VI-RADS score, treatment time, and type of referral hospital were collected. Pathological reports and slides from the referral hospital were ordered and reviewed. The pathological re-evaluation was performed by at least two pathologists at our hospital. Slides that had been used for the initial diagnosis at the referring hospitals were retrieved and re-evaluated at our institution.

We performed descriptive statistics and investigated the following: (1) concordance rate of pathological staging between our hospital and local hospitals, (2) factors influencing discrepancies in pathological stages, (3) treatment changes according to pathology re-evaluation, and (4) the prognosis of patients with a discordant pathology.

This study was conducted in accordance with the principles of the Declaration of Helsinki and was approved by the Internal Review Board of the Tsukuba University Hospital (approval number: H29-030). Informed consent was obtained by the opt-out method through the hospital website. Patients who opted out of the study were excluded.

## Results

### Concordance rates of pathological staging between our hospital and other hospitals

**Fig. 1:**
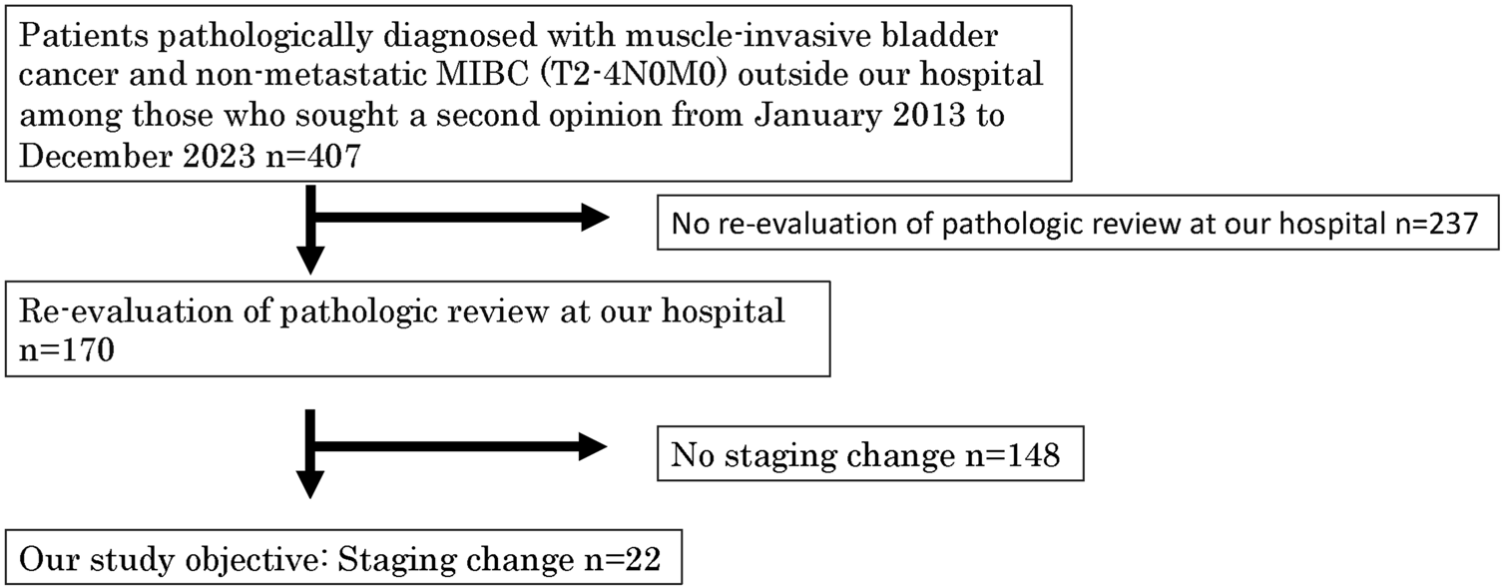
Eligibility flowchart

During the study period, 407 patients were diagnosed with MIBC based on pathology at other institutions and sought consultation at our hospital's outpatient department for a second opinion. Of the 407 patients who visited for a second opinion, 170 chose to continue care at our hospital. The remaining patients likely continued treatment at their original institutions due to geographic, social, or relational factors, although these reasons were not uniformly documented. These 170 patients desired evaluation and treatment at our hospital and underwent re-examination of their pathological diagnosis obtained from the referral hospital. Muscle invasion was not determined, and the pathological stage changed in 22 (12.9%) patients during pathological re-examination (Fig. 1).

All cases were histologically diagnosed as urothelial carcinoma, and no variant histologies. Six patients were diagnosed with Ta. Among them, three had muscularis propria in their samples, two did not, and in one case, the sampling was uncertain. In addition, 16 patients were diagnosed with T1, of which nine had muscularis propria, two did not, and five had uncertain sampling. In the five patients who had uncertain sampling, the pathologists had difficulty distinguishing between the muscularis propria and muscularis mucosae, with uncertainty or significant crush artifacts, which made it unclear whether the muscularis propria was present (Table 1).

**Table 1:**
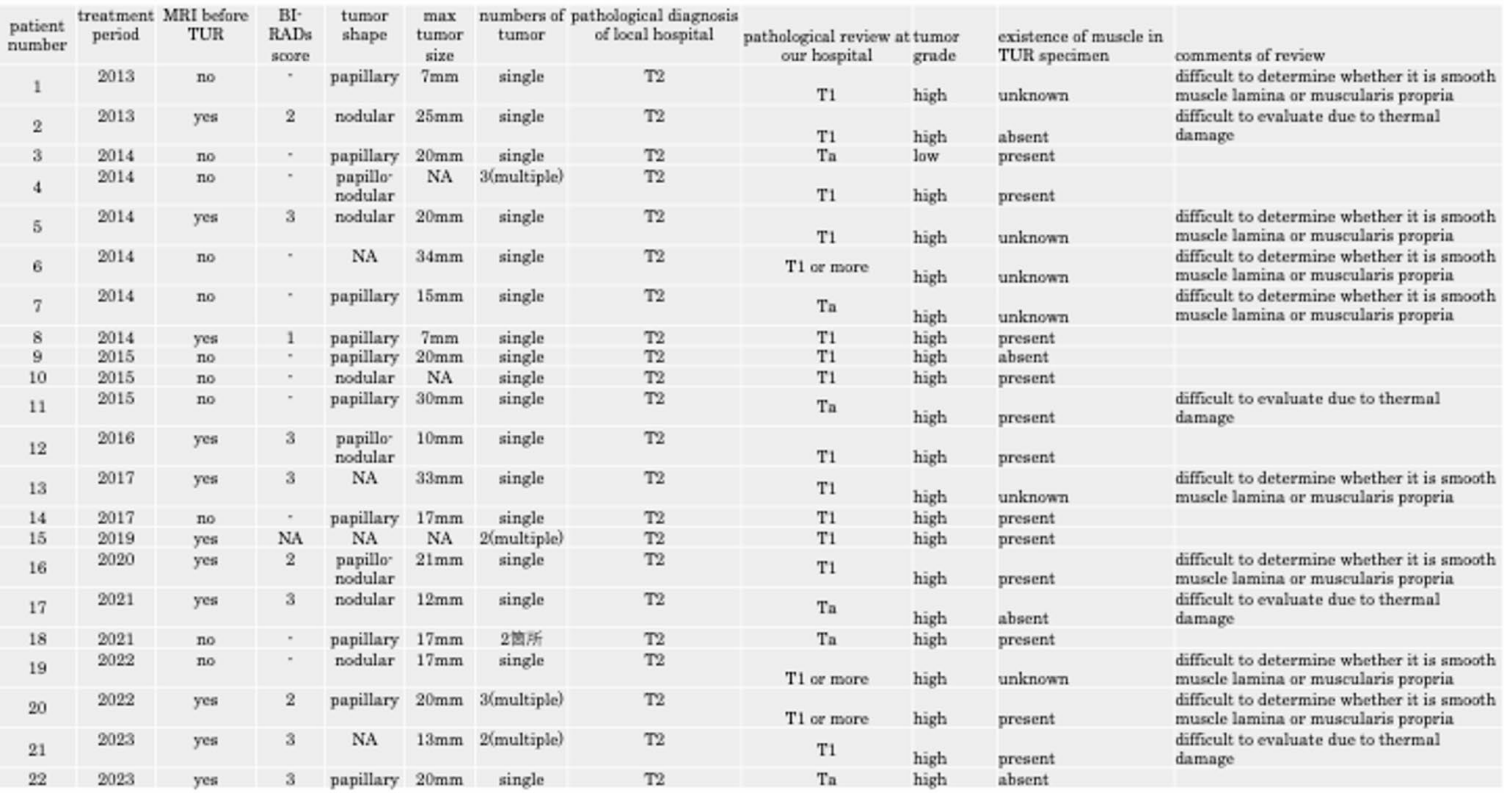
Tumor characteristics of patients with stage changes

MRI was performed before TUR in 11 of the 22 patients. In the 10 cases for which MR images were available, vesical imaging reporting and data system (VI-RADS) evaluation showed that one patient had a score of 1, three had a score of 2, and six had a score of 3. None of the patients had VI-RADS scores of 4 or 5. Twenty-two patients had solitary tumors measuring < 2 cm (Table 1).

### Factors influencing discrepancies in the pathological staging

Factors influencing discrepancies in pathological staging were examined in terms of treatment time and referral hospital type. When divided into two treatment periods, 2013–2016 and 2017–2023, the rates of discrepancy in pathological stage were 14% (11/80) for 2013–2016 and 12.2% (11/90) for 2017–2023. Regarding the type of referral hospital, the discrepancy rate for the pathological stage in patients referred from university hospitals or cancer centers was 6.9% (4/58), whereas that in patients referred from community hospitals was 16.0% (18/112).

### Treatment changes according to the re-evaluation of pathology

Of the 22 cases with discordant results, immediate radical cystectomy was proposed for 4 patients, with 3 patients undergoing treatment at our hospital and 1 receiving treatment at the referral hospital. Eighteen patients were re-evaluated using TUR at our hospital (Table 2).

**Table 2:**
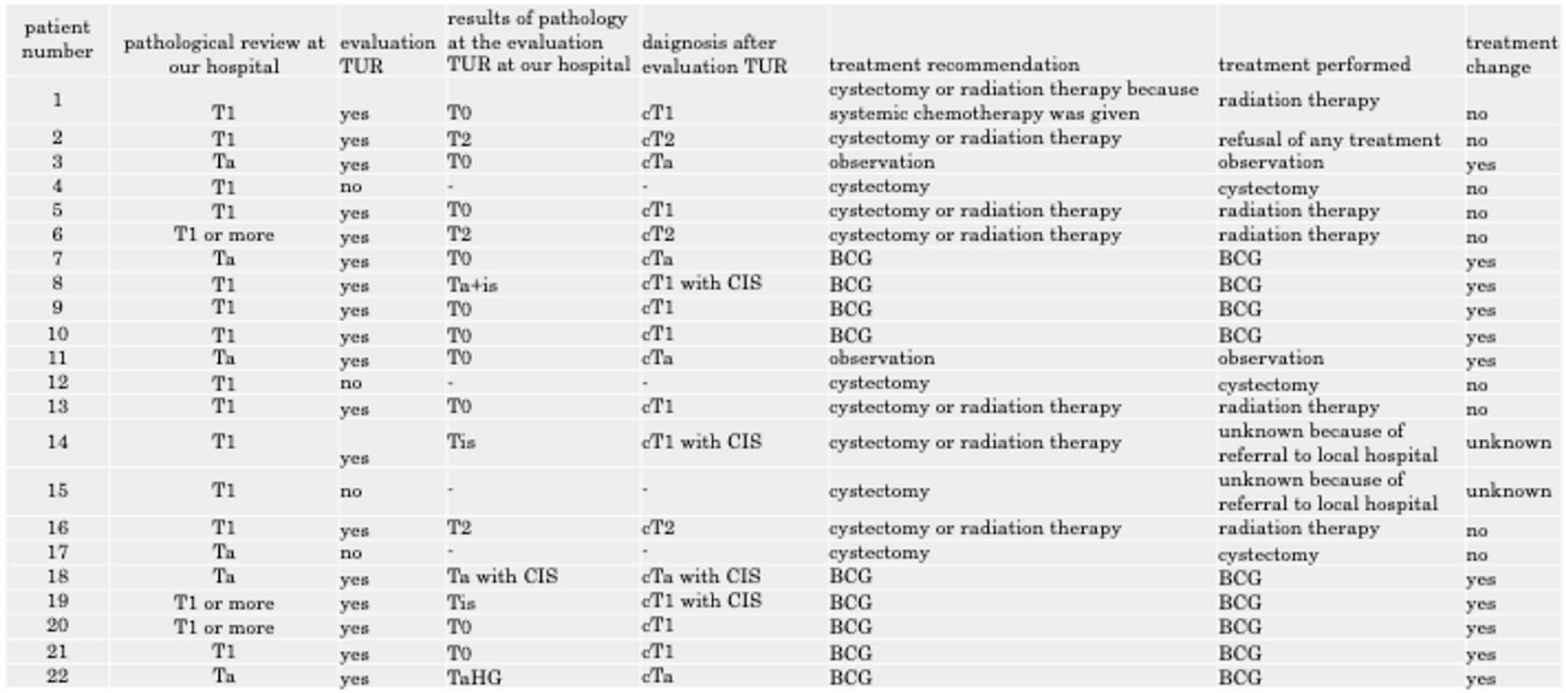
Results of TUR evaluation and treatment change

The pathological results of TUR evaluation were as follows: among the five patients who were pathologically re-evaluated as having Ta, the results of TUR evaluation were as follows: three patients with T0, one patient each with Ta and Tis, and none with T2 or T13. Among the seven patients who were pathologically re-evaluated as having T1 or higher (without muscularis propria sampling or uncertain), the results of TUR evaluation were as follows: four patients with T0, two with T2, one with Tis, and none with T1 or Ta. Among the six patients who were pathologically re-evaluated as having T1 (with muscularis propria sampling), the results of TUR evaluation were as follows: three patients with T0, two with Tis, one with T2, and none with T1 or Ta (Table 2).

The pathological results of the TUR evaluation led to the following treatment recommendations: radical cystectomy was offered to one patient, radiation therapy to six, intravesical Bacillus Calmette-Guérin (BCG) instillation to nine, and observation to two. Eleven patients were treated for NMIBC, and 6.5% (11/170) required treatment modifications.

### Prognosis of patients with pathologically discordant cases

Of the 11 patients who underwent radical cystectomy or radiation therapy (4 patients without TUR evaluation and 7 with TUR evaluation), during a median follow-up period of 41 months (range, 2–113 months), 9 had no evidence of disease, and 2 had distant metastatic recurrence (Table 3).

**Table 3:** Prognosis of patients with treatment changes

Patient number	Treatment performed	Follow-up period (months)	Recurrence	Detailed information of recurrence
3	Observation	55	Yes	Intravesical recurrence (pTa)
7	BCG	72	No	
8	BCG	1	No	
9	BCG	102	Yes	Intravesical recurrence (pTa)
10	BCG	49	No	
11	Observation	54	No	
18	BCG	36	No	
19	BCG	18	Yes	Metastatic recurrence without intravesical Recurrence
20	BCG	2	No	
21	BCG	9	No	
22	BCG	6	No	

Of the nine patients who received BCG therapy and two patients who underwent observation during a median follow-up period of 36 months (range, 1–102 months), eight patients had no evidence of disease, two had Ta intravesical recurrence, and one had lung metastasis despite no intravesical recurrence.

## Discussion

In this study, we observed a discrepancy rate of 12.9% (22/170) in the pathological reassessment of muscular layer invasion among patients pathologically diagnosed with MIBC at the referral hospitals. Among the 22 patients who were downstaged, 18 underwent TUR evaluation and 15 were diagnosed with NMIBC. Of these, 11 were treated with BCG or observed. One patient showed distant metastasis. However, among the remaining 10 patients, there was no progression to MIBC, and our treatment strategy was considered appropriate.

Several problems associated with TUR have been identified as factors that influence pathological discrepancies [7]. The reasons for understaging include piecemeal tumor sampling, thermal artifacts, and inadequate muscular layer sampling. It should also be noted that variations in TURBT technique among different surgeons or institutions may have influenced the pathological interpretation due to differences in resection depth or sampling quality. Although we could not evaluate surgical factors in detail in this study, such variability should be addressed in future prospective analyses. Conversely, the reasons for overestimation include mistaking intrinsic muscular hypertrophy or thermal artifacts for muscular layer invasion.

In this study, although the muscular layer was present in 12 of the 22 patients reviewed at our institution, muscular layer invasion was not evident. Additionally, in 4 of the remaining 10 patients, the muscular layer was not sampled, and in 6 patients, it was difficult to distinguish between thermal artifacts or intrinsic muscular layer versus lamina propria invasion, resulting in a diagnosis of at least T1. For these cases with uncertain muscle presence, repeat TURBT was performed to confirm staging before making final treatment decisions. In one such case, although muscularis propria was present, the tumor was clearly confined to the mucosa, and thus the diagnosis was revised from T2 to Ta. This highlights the potential for overdiagnosis due to thermal artifact or muscle hypertrophy and the value of multi-pathologist review.

MRI is also a useful supportive diagnostic tool for bladder cancer staging [6], although the presence or absence of muscle invasion in pathological diagnoses is important. In our study, VI-RADS scores were used in an exploratory manner to assess whether imaging might support or correlate with pathological findings. The tumor characteristics of the cases with staging discrepancies in our study were small tumor size, solitary lesions, and VI-RADS score < 3. In such cases, it is necessary to keep in mind the possibility of pathological overdiagnosis, and it is worth considering confirming and discussing the pathological diagnosis with the pathologist who made the diagnosis.

Factors influencing pathological stage discrepancies were examined with respect to the treatment period and the type of referral hospital. We hypothesized that with the increasing standardization of muscular layer sampling in recent years, the rate of pathological discrepancies would decrease and the frequency of pathological stage discrepancies would not vary by the treatment period. When dividing the types of referral hospitals into university hospitals/cancer centers and community hospitals, the pathological discrepancy rate was 6.9% (4/58) for patients referred from university hospitals/cancer centers compared to 16.0% (18/112) for those referred from community hospitals, showing an approximately 10% higher rate for community hospitals.

The literature regarding inter-pathologist discrepancies in pathology mainly focuses on NMIBC [10, 11]. Studies targeting T1 lesions showed rates of upstaging to T2 ranging from 0 to 17% and those of downstaging to Ta ranging from 15 to 55% [11]. Studies evaluating a subset of patients with MIBC showed downstaging rates of 10/31 [9] and 1/55 [14]. Although there are no reports specifically targeting only patients with MIBC. Despite the increasing recognition of the importance of muscular layer sampling in staging, there were instances where confirmation of muscular layer sampling was not obtained, even when patients were diagnosed with T2.

This study has several limitations, including its retrospective nature, inclusion of only patients who desired treatment at our institution, and evaluation by pathologists at our institution. In the future, the standardization of diagnoses may lead to an increase in the concordance rate among diagnostic institutions and ultimately allow for appropriate medical care for patients with MIBC.

## Conclusions

In conclusion, the inter-pathological agreement rate for patients diagnosed with MIBC was high (12.9%). It is important not to blindly accept the diagnosis of MIBC from another institution but rather to consult on treatment strategies after pathological re-assessment.

## Data Availability

No datasets were generated or analysed during the current study.
